# The Role of microRNA-23a-3p in the Progression of Human Aging Process by Targeting FOXO3a

**DOI:** 10.1007/s12033-023-00746-7

**Published:** 2023-04-23

**Authors:** Shan Wang, Ying Sun, Lan Yao, Yunli Xing, Huayu Yang, Qing Ma

**Affiliations:** Department of Geriatrics, Beijing Friendship Hospital, Capital Medical University, Beijing, 100050 China

**Keywords:** Human aging, miR-23a, Proliferation, FOXO3a, WI-38 cell

## Abstract

Aging results in deterioration of body functions and, ultimately, death. miRNAs contribute to the regulation of aging. The aim of this study was to explore the contribution of miRNAs to aging and senescence-related changes in gene expression. The expression changes of miRNAs in the blood of people and animal samples collected from different age subjects were examined using Affymetrix miRNA 4.0 microarray and qRT-PCR. MTT assay and flow cytometry were used to examine the effect of miR-23a on cell functions in WI-38 cells. The expression levels of 48 miRNAs, including miR-23a, miR-21, and miR-100, in the blood samples were higher in the middle-aged group than in the young or elderly group. Animal studies further suggested that the expression of miR-23a increased with age. In addition, upregulation of miR-23a dramatically suppressed the cell proliferation and arrested the WI-38 cell cycle in vitro. FOXO3a has been identified as a target gene of miR-23a. MiR-23a downregulated the expression of FOXO3a in WI-38 cells. MiRNAs have different expression levels in different age groups. miR-23a could suppress cell proliferation and arrest the cell cycle in WI-38 cells, which elucidated the mechanism through which miR-23a exerts pivotal role in WI-38 cells by targeting FOXO3a.

**Supplementary Information:**

The online version contains supplementary material available at 10.1007/s12033-023-00746-7.

## Introduction

Aging is a multifactorial, systemic normal physiological process characterized by progressive degeneration, functional incapacitation, and decreased repair capacity of tissues and organs. Loss of functional integrity increases the risk for multiple age-related diseases, including cardiovascular disease, neurodegenerative diseases, osteoporosis, sarcopenia, and cancer [1, 2]. With the gradual increase in the global population, researchers have shown a growing interest in exploring the molecular mechanisms of aging and related pathologies [3]. Normal human fetal lung fibroblasts (WI-38) are among the most common cell models used to study cellular aging and senescence [4]. Exploring WI-38 in vitro and in vivo can further the understanding of human aging mechanisms and provide a basis for well-being and longevity.

MicroRNAs (miRNAs) are a class of small non-coding RNA molecules (19–22 nucleotides) that are important for regulating protein expression through inhibiting translation or inducing mRNA degradation by binding to the 3’-untranslational region (3’-UTR) of target mRNAs [5–7]. miRNAs are important in regulating longevity and canonical aging pathways [8, 9]. In 2013, Boon et al*.* discovered that high expression of miR-34a can induce age-related cardiomyocyte death and decreased cardiac contractile function [10]. Besides, miR-71 and miR-239 affect lifespan through insulin/IGF-1 and DNA damage checkpoint pathways [11, 12]. Therefore, studying the regulatory role of miRNAs in human aging can help to elucidate their potential therapeutic applications. However, the contribution of miRNAs to aging and senescence-related changes in gene expression remains elusive. Thus, this study aimed to explore the contribution of miRNAs to aging and senescence-related changes in gene expression.

## Materials and Methods

### Cell Culture

Normal human fetal lung fibroblast WI-38 cells were purchased from the Cell Bank of National Infrastructure of Cell Line Resource (Beijing, China). The cells were cultured in DMEM with 10% FBS and 1% penicillin/streptomycin in a humidified atmosphere containing 5%CO_2_/95% air at 37ºC. Cells passaged 10–25 times were used to avoid replicative senescence; WI-38 cells have a mean life span of about 45–60 passages.

The present study was approved by the Ethics Committee of our hospital (No. ***). All participants provided written informed consent.

### Blood Samples

Forty-three samples of blood were obtained from male patients treated at the Department of Geriatrics of our hospital. The inclusion criteria were: (1) age group: youth group (20–35 years old), middle-aged group (45–60 years old), older adult group (70–90 years old); (2) patients who underwent health check-ups in the Department of Geriatrics of our hospital. The exclusion criteria were: (1) patients who have been using > 3 drugs over an extended period of time; (2) patients with carcinoma, myelodysplastic syndrome, leukemia, lymphoma, or purpura; (3) patients with cirrhosis, severe infection, or other organ’s dysfunction.

In addition, blood samples from male C57BL/6 mice of different ages were obtained from the Beijing Vital River Laboratory Animal Technology Co. After using isoflurane anesthesia, the blood (700–900 µL) was collected from the retro-orbital plexus, placed into sterile tubes, and analyzed according to blood specification.

Written informed consent was obtained from all the samples. All specimens were handled and made anonymous according to ethical and legal standards. All details are listed in Table 1.

**Table 1:** Demographic information in the 3 groups

Microarray	Young	Middle age	Old age
Number	2	2	2
Age	30	57.5	87
Race	China	China	China
Sex	Male	Male	Male
Tumor	No	No	No
CVD	No	No	1

(B) Validation	Young	Middle age	Old age
Number	12	13	12
Age	32.8 ± 3.8	52.8 ± 3.8	82.2 ± 4.4
Race	China	China	China
Sex	Male	Male	Male
Tumor	No	No	No
CVD	No	2	5
Hypertension	No	2	6

*CVD* cardiovascular disease

### Construction of Recombinant Expression Vectors and Transfection

In order to construct the luciferase reporter plasmid, the FOXO3a 3′-UTR was cloned into a dual-luciferase vector psiCHECK-2 (Promega, USA). The primers are shown in Table 2. Briefly, WI-38 cells were transiently transfected with miR-23a mimic, negative control mimics (NC), anti-miR-23a, or anti-NC (RIBOBIO, Guangzhou, China) using Invitrogen™ Lipofectamine 2000 (Life Technologies, New York, USA), according to the manufacturer’s recommendations. After 24 to 48 h post-transfections, cells were used for subsequent experiments.

**Table 2:** Primer sequences used for amplification

Name	Usage	Sequence (5′–3′)
miR-23a	Forward	GCGATCACATTGCCAGGGA
	Reverse	GTGCAGGGTCCGAGGT
	RT-primer	GTCGTATCCAGTGCAGGGTCCGAGGTATTCGCACTGGATACGACGGAAATC
miR-21	Forward	GCGTAGCTTATCAGACTGATGTTG
	Reverse	GTGCAGGGTCCGAGGT
	RT-primer	GTCGTATCCAGTGCAGGGTCCGAGGTATTCGCACTGGATACGACTCAACA
miR-221	Forward	GAGCTACATTGTCTGCTGGGT
	Reverse	GTGCAGGGTCCGAGGT
	RT-primer	GTCGTATCCAGTGCAGGGTCCGAGGTATTCGCACTGGATACGACGAAACC
miR-100	Forward	GCCGCAACCCGTAGATCCG
	Reverse	GTGCAGGGTCCGAGGT
	RT-primer	GTCGTATCCAGTGCACGCTCCGAGGTATTCGCACTGGATACGACCACAAG
miR-128	Forward	CGCGCTCACAGTGAACCG
	Reverse	GTGCAGGGTCCGAGGT
	RT-primer	GTCGTATCCAGTGCAGGGTCCGAGGTATTCGCACTGGATACGACAAAAGA
U6	Forward	GCGCGTCGTGAAGCGTTC
	Reverse	GTGCAGGGTCCGAGGT
	RT-primer	GTCGTATCCAGTGCAGGGTCCGAGGTATTCGCACTGGATACGACAAAATATG
FOXO3a	Forward	TGCCAGGCTGAAGGATCACT
	Reverse	GGGATTCACAAAGGTGTTAAGCTG
GAPDH	Forward	GGTCATCCATGACAACTTTGG
	Reverse	GGCCATCACGCCACAG

### Cell Proliferation Analysis

Cell proliferation was assessed with the MTT assay kit (Dojindo, Tokyo, Japan). The cells were transfected and then plated in a 96-well microplate (Corning Incorporated, New York, USA) 6 h later. They were subsequently incubated at 37ºC in 5% CO_2_ for 24, 48, 72, and 96 h (all experiments were performed in triplicates). At each time point, 20 μl of sterile MTT dye (5 mg/mL) was added to each well and incubated for 4 h at 37 °C. After removal of the medium, 150 μl of DMSO was added to each well and properly mixed for another 10 min. The absorbance at 490 nm was determined using a microplate reader (Thermo Scientific Microplate Reader). IC50 values were calculated from the linear regression of the plot.

### Cell Cycle Assay

After 48 h of transfection, 1 × 10^5^ WI-38 cells were collected and fixed in 75% ethanol overnight at -20˚C. The cells were treated with RNase A (100 ng/mL) for 30 min and stained with PI (50 ng/mL) for 15 min. After staining, the samples were measured with an EPICSⓇ XL (COULTERⓇ) and analyzed using an EXPO 32ADCXL (COULTERⓇ). The percentages of cells in the G0/G1, S, and G2/M phases of the cell cycle were determined using Multicycle for Windows 32-bit.

### Dual-Luciferase Reporter Assay

WI-38 cells were cotransfected with miR-23a mimics, NC mimic, anti-miR-23a, anti-NC, and the pLuc/pLuc-FOXO3a plasmid. Luciferase activity was determined after transfection for 24 h using a Dual-Luciferase Reporter Assay System (Promega, USA) according to the manufacturer’s instructions. The luciferase activity was normalized to the Renilla luciferase activity as an internal standard.

### RNA Extraction and Quantitative Real-Time PCR

Total RNA was extracted using TRIzol (Invitrogen, USA) according to the manufacturer’s instructions. In order to quantify the expression of miRNA, the cDNA was reverse-transcribed using a cDNA synthesis kit (TransGen Biotech, China). The RT-PCR was performed using an SYBR PrimeScript miRNA RT-PCR kit (Takara, Japan) on an ABI 7500 Fast Real-Time PCR system (Applied Biosystems). The miRNA levels were tested with Taqman MicroRNA Assay (Applied Biosystems). U6 and 18S rRNA were used as the endogenous controls for miRNA and mRNA. Small RNA was used as an internal control. The primers have been reported before [13] and are shown in Table 2.

### Western Blot

A RIPA lysis buffer (CWBIO, Beijing, China) was used to extract the total proteins. The protein concentration was determined using the Bradford assay. Proteins were separated with 10% SDS-PAGE (Beyotime Institute of Biotechnology) at 60 V for 30 min and then transferred onto PVDF membranes (Millipore, USA). Subsequently, the PVDF membrane was incubated with an anti-FOXO3a antibody (1:100) and anti-GAPDH antibody (1:100) at room temperature for 3 h, followed by secondary antibodies horseradish peroxidase-conjugated Ig (1:10,000) at room temperature for 1 h. Chemiluminescent detection was visualized by ECL Detection Reagents (Amersham Bioscience). The relative protein expression was measured using Image-Pro Plus 6.0 software. Data were presented as the density ratio compared with GAPDH.

### Statistical Analyses

Statistical analyses were performed using SPSS v.19.0 software, and graph presentations were completed using GraphPad Prism 8 Software. All data were presented as means ± standard deviation (SD). Comparisons between the two groups were evaluated by Student’s *t* test. *P* < 0.05 was considered statistically significant.

## Results

### Microchip Analysis of miRNAs Involved in Human Aging

To clarify the changes in miRNA expression levels in different age groups, we collected peripheral blood samples of people aged 27–37 (youth group), those aged 45–58 (middle-aged group), and those aged 75–88 (older adult group). Two blood samples from each group were selected for miRNA chip analysis, followed by 37 samples for verification. The demographic information is shown in Table 1.

**Fig. 1:**
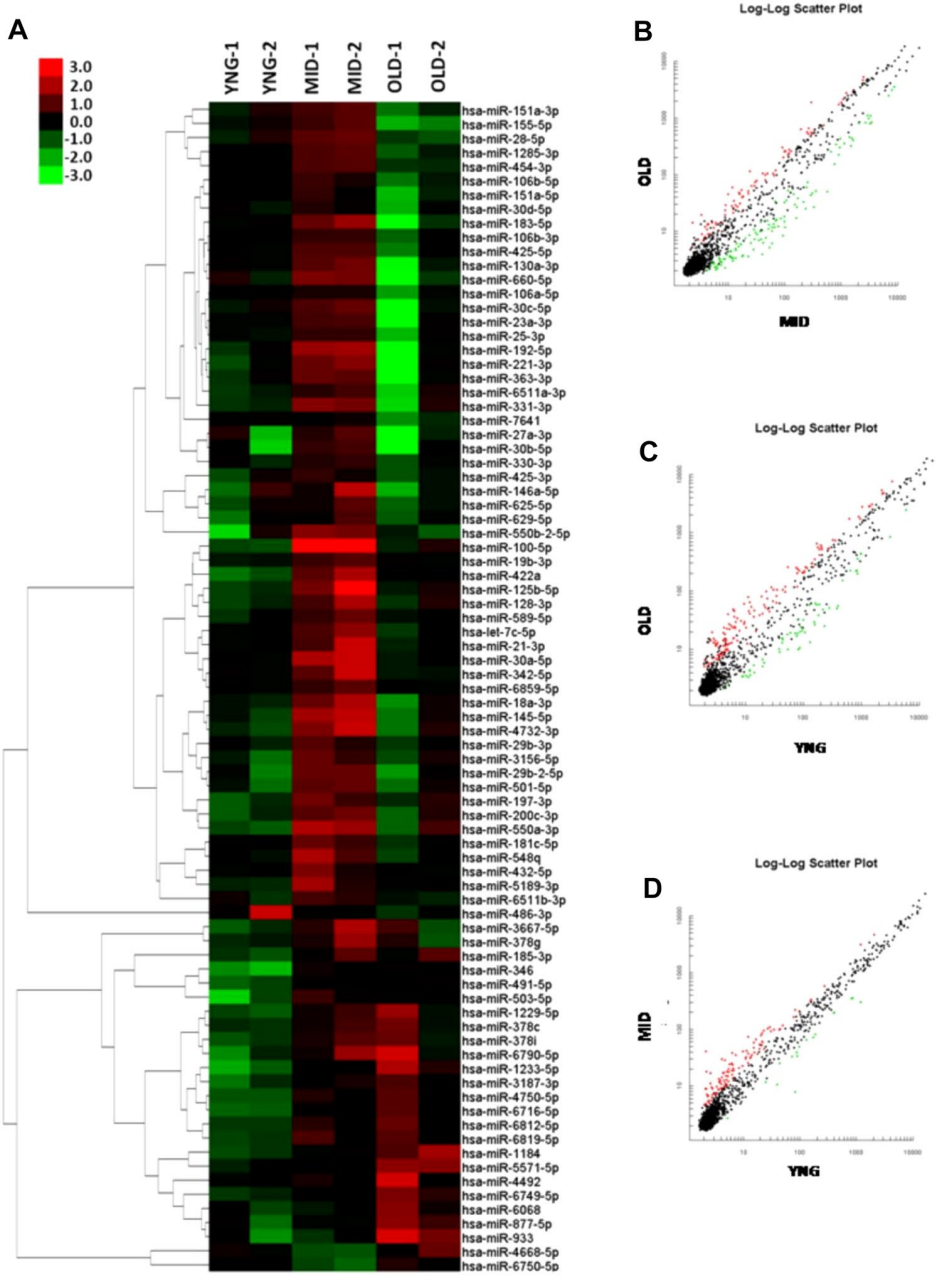
miRNA expression levels in different age groups. **A** Affymetrix miRNA 4.0 chip technology was used to analyze miRNA expression in 2 young subjects (YNG), 2 middle age subjects (MID), and 2 old age subjects (OLD); cluster software was used to form a heat map. **B** Scatter plot: the content of miRNAs in the older adult group vs. middle-aged group **C** Scatter plot: miRNAs in the older adult group vs. young group. **D** Scatter plot: the content of miRNAs in the middle-aged group vs. young group. Green dots represent downgrades more than two times, while red dots represent downgrades more than two times

We used the Affymetrix miRNA 4.0 microarray technology to analyze the expression changes of 4757 miRNAs. The heatmap showed the differential expressed miRNAs in the three groups (Fig. 1A). The expression levels of miRNAs were significantly increased in middle-aged populations but significantly decreased in the older adult and young populations (all *P* < 0.05). When we compared these three sets of results pair-wise, we found that the expression level of miRNAs was still highest in the middle-aged group (Fig. 1B–D). These results suggest that various miRNAs are involved in human aging, which provides a basis for studying miRNAs in the process of aging.

### The miRNAs Expression Level in the Middle-Aged Group was Higher in the Blood Samples

**Fig. 2:**
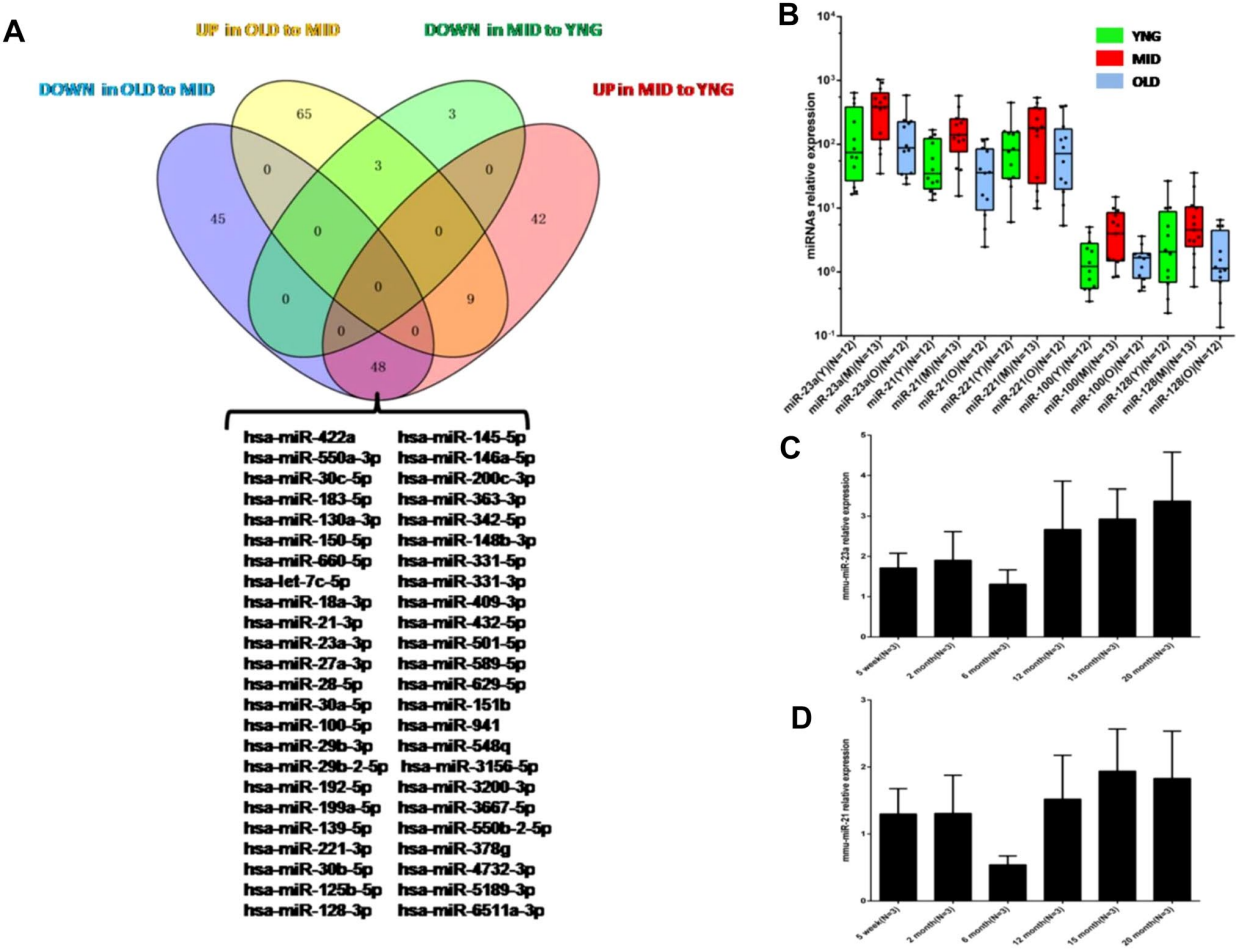
The expression of miRNAs in young groups, middle-aged, and older adults. **A** Up: Using the Venn diagram, the expression of miRNAs in the older adult group was more than 2 times higher than that in the middle-aged group (Up in OLD to MID), and the expression level of the older adult group was less than 2 times lower than that in the middle-aged group (Down in OLD to MID), and the expression level of the middle-aged group was more than 2 times higher than that of the young group (Up in OLD to MID). MID to YNG) and miRNAs whose expression level was less than 2 times (Down in MID to YNG). Down: List 48 miRNAs as overlapping miRNAs in the two sets of data, with the expression level of the older adult group being less than 2 times that of the middle-aged group (Down in OLD to MID) and the expression level of the middle-aged group being more than 2 times that of the young group (Up in MID to YNG). **B** The relative expression levels of miR-23a, miR-21, miR-221, miR-128, and miR-100 in 12 young subjects (YNG), 13 middle-aged subjects (MID), 12 old age subjects (OLD), detected by qRT-PCR. The middle line in the box plot is about the median of the data. *P value for interaction between three different age groups < 0.05; **P value for interaction between three different age groups < 0.01. **C** and **D** qRT-PCR showing relative expression levels of miR-23a or miR-21 in blood samples of C57 mice aged 5 weeks, 2 months, 6 months, 12 months, 15 months, and 20 months

Through microchip analysis, we found that the expression levels of 48 miRNAs were significantly increased in the middle-aged group and significantly decreased in the older adult and young groups (Fig. 2A). Changes in miRNA expression multiples are shown in Supplemental Table 1

Five miRNAs (i.e., miR-23a, miR-21, miR-221, miR-100, and miR-128) met the following criteria: (1) significantly increased in the middle-aged group and significantly decreased in the older adults and young groups; (2) yielded a ratio between two examine groups, which was below or around 0.3 (for up-regulated miRNAs) or above 2.0 (for downregulated ones); (3) those miRNAs that have been reported that might be related to aging, and were selected based on the microchip analysis results. Then, qRT-PCR was used to detect the expression mRNA levels of these five miRNAs in different age groups. The results showed that compared with young-aged or old-aged groups, the relative expression of miR-23a, miR-21, and miR-100 in the middle-aged group was significantly increased (Fig. 2B; Supplemental Table 2).

Meanwhile, we also detected the expression levels of miR-23a and miR-21 in mice of different age (5 weeks and 2, 6, 12, 15, and 20 months). The results showed that miR-23a and miR-21 increased with age (Fig. 2C, D).

### Upregulation of miR-23a Suppresses Cell Proliferation But Arrests Cell Cycle in WI-38 Cell Line

**Fig. 3:**
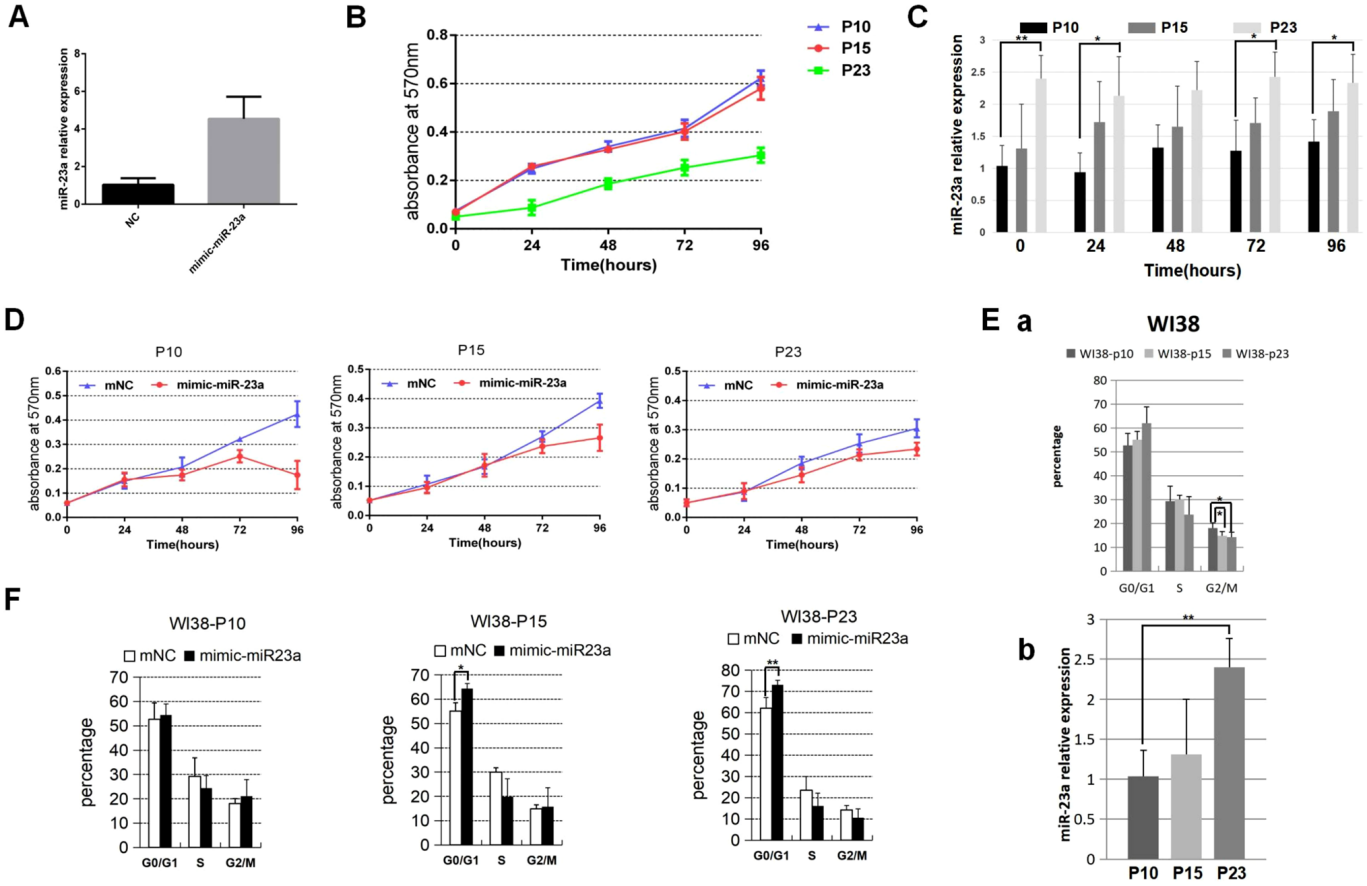
MiR-23a could inhibit cell proliferation and promote cell cycle progression in WI-38 cells. **A** Upregulation of miR-23a following transfection with 50 nM miR-23a mimic or NC in WI-38 cells. **B** The proliferation of different generation WI-38 cell lines (P10, P15, and P23) was analyzed by the MTT assay. **C** The relative expression of miR-23a in the proliferation of WI-38 different generations (P10, P15, and P23) was detected by qRT-PCR. **D** The proliferation of different generations of WI-38 cell lines (P10, P15, and P23) transfected with miR-23a mimic. **E** a. The cell cycle distributions of different generations of WI-38 cell lines (P10, P15, and P23) analyzed by flow cytometry. b. The relative expression of miR-23a in different generations of WI-38 cell lines (P10, P15, and P23) was detected by qRT-PCR. **F** The cell cycle distributions of different generations of WI-38 cell lines (P10, P15, and P23) transfected with miR-23a mimic and detected by flow cytometry; the test was repeated four times (*P < 0.05, **P < 0.01)

In order to investigate the role of miR-23a in cell proliferation and the cell cycle of WI-38 cells, miR-23a mimics or negative control (NC) were transfected into WI-38 cells. After 48 h, the expression levels of miR-23a were significantly up-regulated more than 4 times in WI-38 cells transfected with miR-23a mimics (Fig. 3A). Next, the MTT assay was used to detect the proliferation of WI-38 cells passaged for different times (P10, P15, and P23), and simultaneously check the endogenous expression levels of miR-23a. Results showed that the proliferation level of P23 cells was significantly lower than in P10 and P15 cells (Fig. 3B), and the miR-23a expression in P23 cells was significantly up-regulated than in P10 cells (Fig. 3C).

Next, we performed transfection with miR-23a mimics or NC group and examined the proliferation in different generations of WI-38 cells through MTT assay. The result indicated that miR-23a could inhibit the proliferation of different passages (P10, P15, and P23) (Fig. 3D).

Furthermore, we investigated the effect of miR-23a on the WI-38 cell cycle. Flow cytometry was performed on different passages of WI-38 cells, revealing that the endogenous expression levels of miR-23a increased with the increasing passages of WI-38 cells, especially in P23. Flow cytometry revealed that WI-38 cells in the G2/M phase were inhibited. Next, we transfected miR-23a mimic and NC in different passages of WI-38 cells (Fig. 3E). Flow cytometry results revealed that upregulation of miR-23a dramatically arrested cell cycle progression in different generations of WI-38 cells (P10, P15, and P23) compared to the NC group, in which cells in the S phase were decreased (Fig. 3F). To sum up, this data confirmed that miR-23a could inhibit cell proliferation and cell cycle in the WI-38 cell line.

### miR-23a Targets FOXO3a in a WI-38 Cell Line

**Fig. 4:**
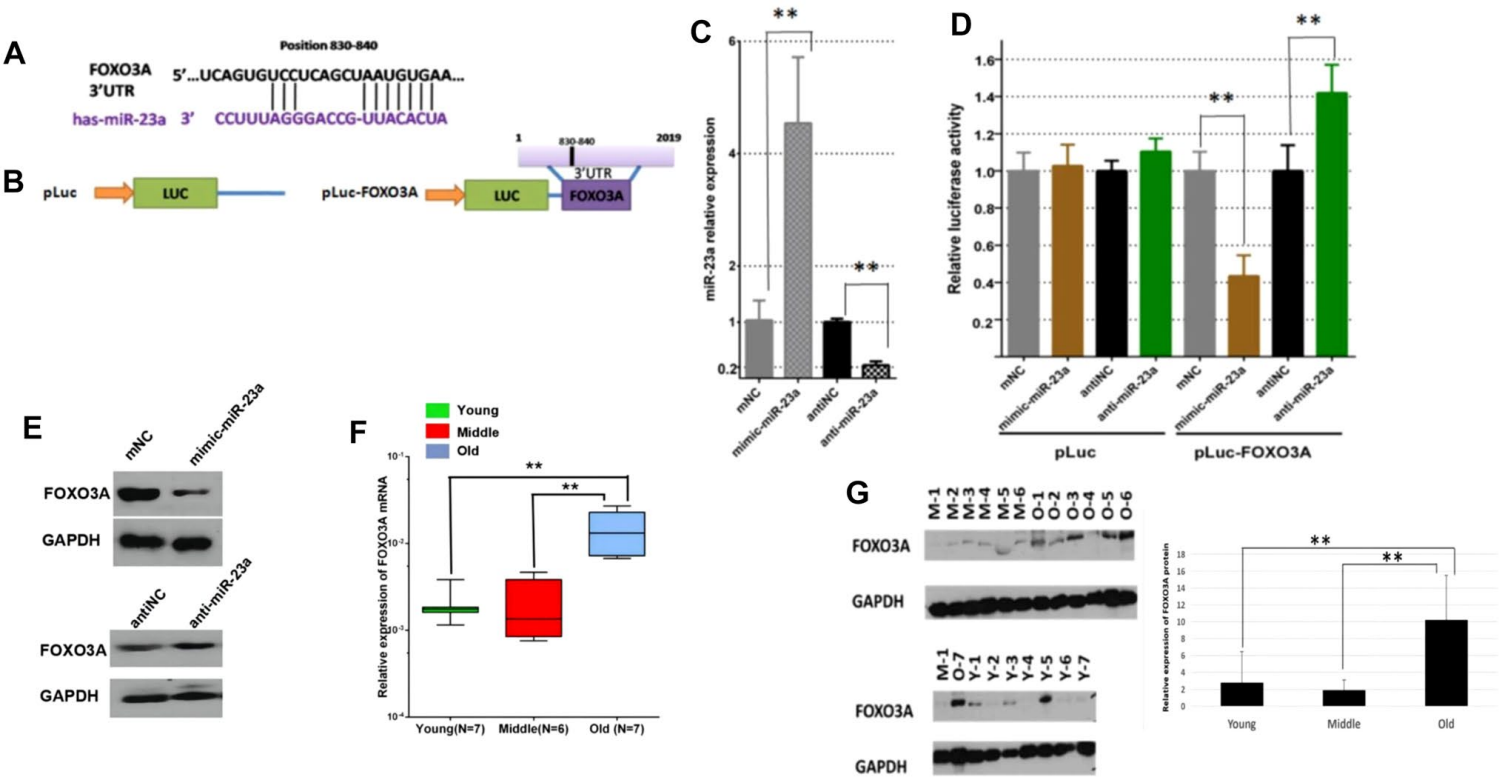
FOXO3a was a target gene of miR-23a in WI-38 cells. **A** FOXO3a 3′-UTR contains predicted miR-23a binding sites. The figure shows the alignment of miR-23a with the FOXO3a 3′-UTR. **B** Luciferase reporter plasmid without or containing FOXO3a 3’UTR region prepared by molecular cloning method. **C** The miR-23a mRNA expression levels in the WI-38 cells transfected with miR-23a mimics or the NC, anti-miR-23a, or the anti-NC detected by qRT-PCR. **D** Dual-luciferase reporter assay. In WI38 cells transfected with pLuc plasmid or pLuc-FOXO3a plasmid, the relative luciferase activity after knockdown or overexpression of miR-23a. **E** The effect of knockdown or overexpression of miR-23a on FOXO3a protein expression in WI-38 cells detected by western blot. **F** The relative expression mRNA level of FOXO3a (normalized to GAPDH mRNA) in different age groups (Young people (Y), Middle age people (M), and Old people (O)) was detected by qRT-PCR. **G** The expression protein level of FOXO3a in different age groups (Young people (Y), Middle age people (M), and Old people (O)) was detected by Western blot. (*P < 0.05, **P < 0.01)

To screen the target genes of miR-23a, we used the TargetScan to predict potential targets. FOXO3a was predicted as a potential target. miR-23a can bind to the 3’UTR region of FOXO3a (Fig. 4A). Meanwhile, luciferase reporter gene plasmid (pLuc and pLuc-FOXO3a) was prepared (Fig. 4B). We performed qRT-PCR to determine the mRNA levels after transfection miR-23a mimics or NC mimic, anti-miR-23a, or anti-NC. The expression levels of miR-23a were significantly up-regulated (more than 4 times) in WI-38 cells transfected with miR-23a mimics, and the expression efficiency of miR-23a after knockdown was < 0.2 times (Fig. 4C). Luciferase reporter assay further revealed a significant decrease in the luciferase activity of the reporter gene in WI-38 cells cotransfected with pLuc-FOXO3a and miR-23a mimic compared to the control (cotransfected with pLuc vector and NC mimic).

Also, co-transfection of anti-miR-23a with pLuc-FOXO3a vector led to significant up-regulation in luciferase activity, compared to cotransfected with anti-NC with pLuc-FOXO3a vector. Inversely, co-transfection of miR-23a mimic with pLuc vector, anti-miR-23a with pLuc vector did not cause any significant change in luciferase activity in WI-38 cells (Fig. 4D). Moreover, over-expression of miR-23a dramatically inhibited FOXO3a protein expression; however, there was no significant change in FOXO3a protein expression between anti-miR-23a and anti-NC (Fig. 4E). In addition, we performed qRT-PCR and Western blotting to determine the mRNA and protein levels of FOXO3a using blood samples from the older adult, middle-aged, and young subjects. Briefly, the levels of FOXO3a mRNA and protein level expression were increased in the older adult group compared with the middle-aged and young people group (Fig. 4F, G).

The present study indicates that miR-23a could bind to the 3’UTR region of FOXO3a to inhibit the expression of FOXO3a.

## Discussion

Aging is a multifactorial process associated with cellular, tissue, organ, and whole-body changes, which might result in dysfunctional and disease pathogenesis [14, 15]. The exact mechanism associated with aging is still not fully understood. In this study, we investigated the role of miR-23a in WI-38 cells in vitro and in vivo. miRNAs have been reported to have a significant role in aging-related diseases [16]. Numerous miRNAs have been shown to affect lifespan through aging pathways, including insulin/insulin-like growth factor (IGF-1) signaling, sirtuin deacetylase, mitochondrial/reactive oxygen species (ROS) signaling, and DNA damage response [1, 17, 18]. A study of *Drosophila* body size showed that miR-8 promotes cell growth to non-autonomously regulate body size cells by targeting USH and inhibiting PI3K in the IIS pathway [19]. Previous studies have shown that the inflammatory mediators interleukins-6 and -8 (IL6 and IL8) are secreted during stress-induced cellular senescence and that miR-146a and miR-146b are up-regulated in senescent primary fibroblasts by targeting and inhibiting these interleukins, thus preventing excessive inflammatory response [20]. In the present study, the expression levels of 48 miRNAs, including miR-23a, miR-21, and miR-100, in the human blood samples were higher in middle-aged subjects than in young and older adults. Also, animal studies suggested that miR-23a and miR-21 increase with age. Accordingly, miR-23a and miR-21 might be related to aging and some aging diseases. Studies have found that the incidence of some cancers, such as brain and nervous system tumors, non-Hodgkin lymphoma, leukemia and similar, increase in the middle-aged population [21]. Also, researchers reported that miR-23a and miR-21 might represent a diagnostic and prognostic markers for these cancers and have an important role as oncomiR [22–27]. Therefore, these miRNAs may be associated with the induction of some high-risk diseases in middle age.

In this study, we further examined the role of miR-23a on aging. miR-23a is important in controlling cell growth and proliferation [28]. The function of miR-23a in body cells can inhibit the proliferation of normal cells but also promote the generation of some diseases, therefore accelerating the aging of the body [29, 30]. Herein, we demonstrated that miR-23a could inhibit cell proliferation and arrest the cell cycle in WI-38 cells, thus showing that miR-23a is pivotal in aging. Combined with the changes in the expression level of miR-23a in different age groups, the increased expression level of miR-23a can have an important role in cell senescence and suppress cell proliferation. Therefore, the function of miR-23a in body cells can inhibit the proliferation of normal cells and may also promote the generation of some age-related diseases, which commonly begin to occur in middle age, such as coronary artery disease, diabetes mellitus, brain tumor, and similar [21, 22, 31–33].

FOXO3a is a member of the FOXO subfamily of forkhead transcription factors, which mediate various cellular processes, including apoptosis, proliferation, and cell cycle progression [34–37]. Interestingly, recent studies have discovered that FOXO3a could take part in an autophagy program to protect cells from environmental stresses [38, 39]. Since 2008 and 2009, Willcox and Flachsbart confirmed that FOXO3a is widespread in people > 90 years old and is closely related to longevity [40–42]. Accordingly, FOXO3a has been regarded as a longevity gene that is closely associated with aging. In addition, FOXO3a can represent an important target to inhibit cancer cell progression, linked to many age-related diseases, such as breast cancer [43, 44], glioblastoma [45], leukemia [46, 47], and acute myocardial infarction [48], which commonly occur in middle age [21]. At present, some evidence suggests that FOXO3a is regulated by some miRNAs, such as miR-155 and miR-96 [49, 50]. Our data showed that FOXO3a could be a direct target of miR-23a in WI-38 cells at the molecular level. Therefore, it is supposed that miR-23a may bind to FOXO3a 3’UTR to regulate its protein expression, impact some age-related diseases, or participate in the human aging process. This illustrates the mechanism of action of miR-23a and longevity gene FOXO3a in the human aging process, thus laying the foundation for molecular biology research on human aging.

The present study has some limitations. For example, except for miR-23a, other miRNAs need to be further explored. Also, data need to be analyzed in a larger sample size. Moreover, further studies are needed to elucidate the relationship between FOXO3a and miR-23a activity in human aging, such as the function of other molecules downstream of FOXO3a.

In conclusion, our data showed that miR-23a could suppress cell proliferation and arrest cell cycle by targeting FOXO3a in WI-38 cells. Together with recent findings that miR-23a regulates PKCα in ovariectomized and normal aging female mice [51, 52], our findings underscore the importance of miRNAs in regulating normal cell senescence and aging processes.

### Supplementary Information

Below is the link to the electronic supplementary material.

Supplementary file1 (DOCX 25 kb)

## Data Availability

The datasets used and/or analyzed during the current study are available from the corresponding author upon reasonable request.
