# Supplementary material for: The Role of microRNA-23a-3p in the Progression of Human Aging Process by Targeting FOXO3a
Source: Mol Biotechnol. 2023 Apr 23;66(2):277–87. doi: 10.1007/s12033-023-00746-7 (PMC10803409; doi:10.1007/s12033-023-00746-7)
Supplement: Supplementary file 1 — Supplementary file1 (DOCX 25 kb) [file 12033_2023_746_MOESM1_ESM.docx]

**Supplemental Table 1.** Relative expression of 48 miRNAs by microarray

| **ProbeSetID** | **Transcript ID(Array Design)** | **OLD_1** | **OLD_2** | **MID_1** | **MID_2** | **YNG_1** | **YNG_2** | **O to M Ratio** | **M to Y Ratio** |
| --- | --- | --- | --- | --- | --- | --- | --- | --- | --- |
| 20503800 | hsa-miR-432-5p | 2.3902 | 2.167 | 6.5473 | 3.0968 | 2.1444 | 2.3408 | 0.4725 | 2.1502 |
| 20515645 | hsa-miR-3200-3p | 1.8615 | 20.334 | 30.923 | 56.344 | 10.465 | 12.675 | 0.2543 | 3.7712 |
| 20511563 | hsa-miR-548g | 2.4298 | 4.606 | 16.773 | 7.8779 | 4.0596 | 3.6389 | 0.2854 | 3.202 |
| 20504322 | hsa-miR-589-5p | 2.4934 | 2.8051 | 4.4409 | 6.7612 | 1.9428 | 3.0275 | 0.473 | 2.2538 |
| 20504379 | hsa-miR-629-5p | 36.704 | 72.626 | 89.694 | 150.44 | 29.457 | 84.847 | 0.4553 | 2.1008 |
| 20501279 | hsa-miR-342-5p | 3.1754 | 3.339 | 7.9958 | 19.513 | 4.2585 | 3.5997 | 0.2368 | 3.5007 |
| 20500189 | hsa-miR-29b-2-5p | 3.17 | 11.885 | 25.831 | 24.494 | 10.091 | 3.9077 | 0.2992 | 3.5948 |
| 20500422 | hsa-miR-30c-5p | 4.2972 | 67.265 | 148.47 | 178.65 | 64.338 | 93.521 | 0.2188 | 2.0722 |
| 20500735 | hsa-miR-130a-3p | 6.1302 | 34.958 | 90.14 | 111.69 | 49.528 | 41.162 | 0.2036 | 2.2255 |
| 20500484 | hsa-miR-221-3p | 3.0536 | 44.312 | 90.497 | 114.11 | 21.386 | 34.605 | 0.2315 | 3.6542 |
| 20500117 | hsa-let-7c-5p | 761.2 | 1263.3 | 2421.6 | 3920 | 1151.7 | 1278.9 | 0.3192 | 2.6091 |
| 20501293 | hsa-miR-331-3p | 4.2006 | 32.587 | 72.046 | 61.096 | 15.862 | 17.378 | 0.2763 | 4.0054 |
| 20500162 | hsa-miR-30a-5p | 6.3194 | 7.4534 | 31.403 | 39.1 | 8.0856 | 6.7608 | 0.1953 | 4.7489 |
| 20500724 | hsa-miR-30b-5p | 3.2544 | 45.574 | 71.115 | 85.1 | 48.936 | 8.8227 | 0.3126 | 2.7046 |
| 20500146 | hsa-miR-23a-3p | 11.907 | 186.68 | 304.47 | 321.73 | 156.24 | 154.85 | 0.3171 | 2.013 |
| 20504431 | hsa-miR-660-5p | 2.1271 | 12.441 | 55.671 | 52.053 | 27.955 | 14.701 | 0.1352 | 2.5254 |
| 20502456 | hsa-miR-409-3p | 2.0597 | 2.3109 | 12.085 | 3.4226 | 1.8836 | 2.9323 | 0.2818 | 3.2201 |
| 20500730 | hsa-miR-125b-5p | 2.8484 | 4.4113 | 9.4557 | 33.555 | 1.968 | 2.5138 | 0.1688 | 9.5967 |
| 20501291 | hsa-miR-148b-3p | 1.9333 | 7.3966 | 10.333 | 8.8627 | 2.8807 | 2.57 | 0.486 | 3.5217 |
| 20500452 | hsa-miR-183-5p | 2.6327 | 13.634 | 49.59 | 73.684 | 19.018 | 24.77 | 0.132 | 2.8152 |
| 20500142 | hsa-miR-21-3p | 2.9536 | 4.0005 | 7.0816 | 19.19 | 3.5678 | 3.8782 | 0.2647 | 3.5282 |
| 20500188 | hsa-miR-29b-3p | 2.0118 | 3.4389 | 6.7138 | 4.5094 | 3.094 | 2.3387 | 0.4857 | 2.0659 |
| 20502122 | hsa-miR-422a | 5.9841 | 5.7194 | 12.241 | 23.129 | 2.2794 | 3.0714 | 0.3309 | 6.6102 |
| 20500778 | hsa-miR-146a-5p | 1.9833 | 7.0435 | 9.4557 | 36.804 | 3.3638 | 13.52 | 0.1951 | 2.7399 |
| 20500755 | hsa-miR-145-5p | 2.6606 | 8.2784 | 25.613 | 31.314 | 5.7054 | 3.9807 | 0.1922 | 5.8771 |
| 20500183 | hsa-miR-100-5p | 2.803 | 5.4517 | 33.13 | 49.6 | 2.2103 | 2.1008 | 0.0998 | 19.19 |
| 20500733 | hsa-miR-128-3p | 2.224 | 4.6225 | 5.8454 | 13.456 | 1.9899 | 2.4897 | 0.3547 | 4.3086 |
| 20506012 | hsa-miR-941 | 3.5888 | 20.837 | 20.413 | 51.434 | 8.3553 | 22.31 | 0.34 | 2.3429 |
| 20500385 | hsa-miR-192-5p | 2.8402 | 24.278 | 89.466 | 98.637 | 17.728 | 29.877 | 0.1442 | 3.9513 |
| 20503877 | hsa-miR-501-5p | 20.167 | 59.384 | 105.05 | 112.2 | 41.857 | 20.591 | 0.3662 | 3.4789 |
| 20500432 | hsa-miR-139-50 | 2.1789 | 2.2914 | 3.8948 | 20.134 | 2.1444 | 2.1288 | 0.186 | 5.6231 |
| 20501292 | hsa-miR-331-5p | 3.1532 | 53.42 | 77.21 | 36.325 | 17.414 | 21.737 | 0.4983 | 2.9 |
| 20500158 | hsa-miR-28-5p | 3.2866 | 2.6771 | 11.453 | 12.706 | 4.0744 | 7.3136 | 0.2468 | 2.1215 |
| 20500399 | hsa-miR-199a-5p | 2.5614 | 2.4561 | 4.8916 | 11.355 | 3.4609 | 2.6947 | 0.3088 | 2.6393 |
| 20501206 | hsa-miR-363-3p | 12.381 | 86.539 | 221.22 | 214 | 61.748 | 109.73 | 0.2273 | 2.5381 |
| 20500157 | hsa-miR-27a-3p | 2.6717 | 21.333 | 37.758 | 57.876 | 37.38 | 6.2214 | 0.251 | 2.1934 |
| 20500133 | hsa-miR-18a-3p | 8.4475 | 32.847 | 63.291 | 115.38 | 23.164 | 19.742 | 0.2311 | 4.1643 |
| 20500782 | hsa-miR-150-5p | 6.296 | 111.41 | 240.42 | 451.9 | 166.8 | 154.52 | 0.17 | 2.1546 |
| 20504325 | hsa-mi-550a-3p | 3.5577 | 14.28 | 31.935 | 27.038 | 4.4872 | 3.8487 | 0.3025 | 7.0745 |
| 20501036 | hsa-miR-200c-3p | 3.3417 | 10.428 | 15.255 | 18.428 | 3.4656 | 5.3582 | 0.4088 | 3.8173 |
| 20504561 | hsa-miR-151b | 5.465 | 60.066 | 119.91 | 81.302 | 46.854 | 39.037 | 0.3257 | 2.3426 |
| 20515575 | hsa-miR-3156-5p | 3.7889 | 9.4581 | 17.272 | 11.637 | 5.7054 | 2.9984 | 0.4582 | 3.3214 |
| 20517921 | hsa-miR-3667-5p | 7.9096 | 2.4639 | 6.4957 | 18.535 | 2.1405 | 3.2481 | 0.4144 | 4.6451 |
| 20518794 | hsa-miR-378q | 6.3154 | 2.2813 | 5.9022 | 15.646 | 3.1534 | 3.6389 | 0.3989 | 3.1725 |
| 20519577 | hsa-miR-4732-3p | 3.8154 | 13.492 | 28.107 | 52.496 | 7.6931 | 5.3216 | 0.2147 | 6.1932 |
| 20520569 | hsa-miR-5189-3p | 2.8633 | 3.5835 | 11.682 | 4.3206 | 2.3119 | 2.2463 | 0.4029 | 3.5108 |
| 20518722 | hsa-miR-550b-2-5p | 20.662 | 12.088 | 79.685 | 66.215 | 4.5895 | 32.731 | 0.2245 | 3.9094 |
| 20525024 | hsa-miR-6511a-3p | 3.677 | 23.645 | 27.856 | 34.106 | 11.751 | 15.189 | 0.4409 | 2.3 |

**Supplemental Table 2.** Relative expression of 5 miRNAs in different age groups

|  | | Young Group (N=12) | Middle Group (N=13) | Old Group (N=12) | P value | | |
| --- | --- | --- | --- | --- | --- | --- | --- |
|  |  |  |  |  | Young to Middle | Young to Old | Middle to Old |
| The relative expression of miRNAs  (mean±SD) | miR-23a | 188.7±221.5 | 436±319.2 | 152.4±158.9 | 0.036* | 0.649 | 0.011* |
|  | miR-21 | 64.54±56.1 | 190.5±155.7 | 46.51±42.85 | 0.014* | 0.386 | 0.005** |
|  | miR-221 | 113.2±120.8 | 208.5±176.3 | 121.1±139 | 0.132 | 0.883 | 0.184 |
|  | miR-100 | 1.824±1.557 | 5.014±4.419 | 1.62±0.9213 | 0.027* | 0.699 | 0.016* |
|  | miR-128 | 5.266±7.617 | 8.414±9.993 | 2.171±2.214 | 0.389 | 0.19 | 0.046* |

*, P value for interaction between three different age groups < 0.05;

**, P value for interaction between three different age groups < 0.01.
